# The Computational, Pharmacological, and Physiological Determinants of Sensory Learning under Uncertainty

**DOI:** 10.1016/j.cub.2020.10.043

**Published:** 2021-01-11

**Authors:** Rebecca P. Lawson, James Bisby, Camilla L. Nord, Neil Burgess, Geraint Rees

**Affiliations:** 1 Department of Psychology, Downing Street, University of Cambridge, Cambridge CB2 3EB, UK; 2 MRC Cognition & Brain Sciences Unit, Chaucer Road, University of Cambridge, Cambridge CB2 7EF, UK; 3 Institute of Cognitive Neuroscience, Queen Square, University College London, London WC1N 3AZ, UK; 4 Institute of Neurology, Queen Square, University College London, London WC1N 3BG, UK; 5 Division of Psychiatry, Tottenham Court Road, University College London, London W1T 7NF, UK; 6 Wellcome Centre for Human Neuroimaging, Queen Square, University College London, London WC1N 3AR, UK

**Keywords:** uncertainty, noradrenaline, Bayesian, computational modeling, pupillometry, anxiety, cardiac, perception, learning, blood pressure

## Abstract

The ability to represent and respond to uncertainty is fundamental to human cognition and decision-making. Noradrenaline (NA) is hypothesized to play a key role in coordinating the sensory, learning, and physiological states necessary to adapt to a changing world, but direct evidence for this is lacking in humans. Here, we tested the effects of attenuating noradrenergic neurotransmission on learning under uncertainty. We probed the effects of the β-adrenergic receptor antagonist propranolol (40 mg) using a between-subjects, double-blind, placebo-controlled design. Participants performed a probabilistic associative learning task, and we employed a hierarchical learning model to formally quantify prediction errors about cue-outcome contingencies and changes in these associations over time (volatility). Both unexpectedness and noise slowed down reaction times, but propranolol augmented the interaction between these main effects such that behavior was influenced more by prior expectations when uncertainty was high. Computationally, this was driven by a reduction in learning rates, with people slower to update their beliefs in the face of new information. Attenuating the global effects of NA also eliminated the phasic effects of prediction error and volatility on pupil size, consistent with slower belief updating. Finally, estimates of environmental volatility were predicted by baseline cardiac measures in all participants. Our results demonstrate that NA underpins behavioral and computational responses to uncertainty. These findings have important implications for understanding the impact of uncertainty on human biology and cognition.

## Highlights

• Propranolol increases the effects of expectations on behavior
• Propranolol reduces volatility-linked learning
• Propranolol reduces the impact of prediction errors and volatility on pupil size
• Systolic blood pressure predicts estimated environmental volatility

## Teaser

Noradrenaline (NA) is hypothesized to play a role in how humans adapt to volatility under conditions of uncertainty. Lawson et al. show that blocking NA attenuates probabilistic learning and the phasic encoding of prediction errors in a volatile environment. Furthermore, blood pressure predicts how volatile the world is estimated to be.

## Introduction

When there is an unexpected change in the state of the world—for instance, your regular morning coffee makes you sick one day—humans must decide whether to update their model of the world (and never drink coffee again) or dismiss the unusual outcome as a one-off. To solve this problem, we must flexibly update our beliefs across time, relying on prior expectations when the environment is stable, and disregarding them to promote rapid learning when the world is volatile. The ability to adapt behavior in the face of such uncertainty requires coordination across complex learning dynamics, central sensory systems, and peripheral physiological states. Difficulties balancing prior expectations against new sensory inputs are hypothesized to underlie many different neuropsychiatric and developmental conditions, such as autism,^1^, ^2^, ^3^, ^4^ psychosis,^5^, ^6^, ^7^, ^8^ anxiety,^9^, ^10^, ^11^ and post-traumatic stress disorder (PTSD).^12^^,^^13^^,^^14^ However, the computational and neurochemical mechanisms of this process in normative learning remain poorly understood.

Seminal theoretical work suggests that when an agent is uncertain about the relationships between sensory states, e.g., when the world is volatile, the top-down effects of prior expectations on cortical processing ought to be suppressed.^15^ This shifts the balance of information processing toward sensory inputs, optimizing learning.^16^ The brain’s neuromodulatory systems have widespread cortical projections, making them ideally placed to ratify sudden shifts in neural gain in response to the volatility of the environment.^17^, ^18^, ^19^ The neuromodulator noradrenaline (NA) has been shown to play a key role in detecting environmental change to facilitate learning. For example, increasing NA in rodents enhances bottom-up, thalamo-sensory processing.^20^^,^^21^ By altering tuning functions in sensory cortex, activation of the noradrenergic locus coeruleus (LC) enhances sensory learning.^22^^,^^23^ In contrast, NA blockade has been shown to impair reversal learning and cognitive flexibility in non-human animals.^24^^,^^25^

Humans adapt their learning in response to volatility,^26^ so-called “meta learning”; this process is altered in both anxiety,^27^^,^^28^ autism,^29^ and psychosis. ^30^ Indirect evidence for the role of the NA system in controlling meta-learning comes from studies using pupil size as an index of LC function.^31^, ^32^, ^33^ It has been shown, for example, that pupil size tracks trial-by-trial changes in learning rate,^34^ prediction error,^27^^,^^29^ and volatility.^27^ However, there is a dearth of studies directly examining the pharmacological effects of NA manipulation on learning in a volatile world (though see Jempa et al.^35^ and Marshall et al.^36^).

**Figure 1:**
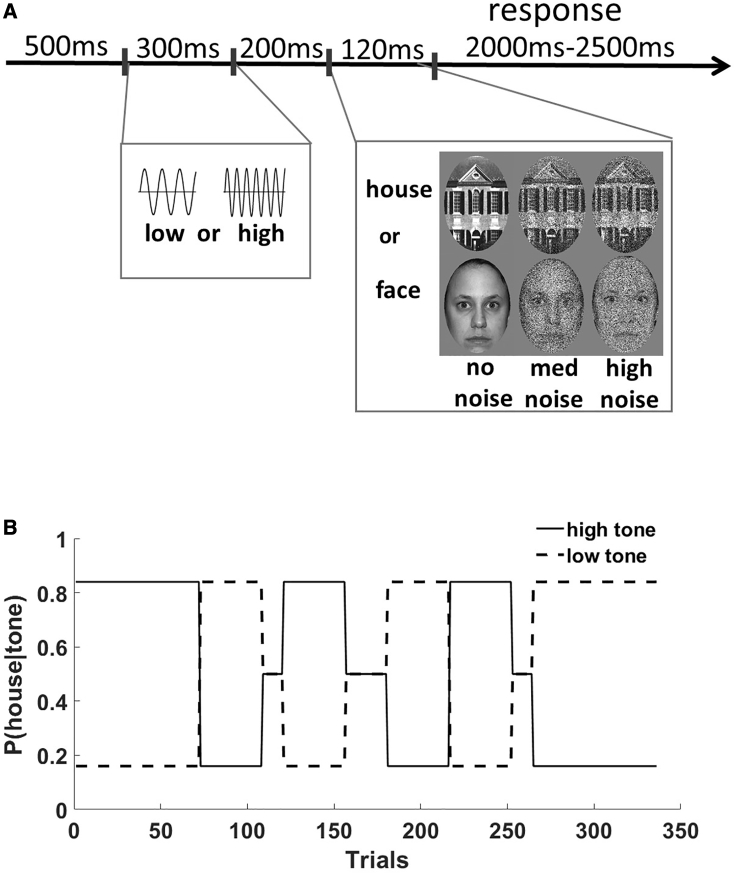
Task Details and Example Trial Structure (A) participants first heard a tone (high or low) followed by a picture (face or house). The pictures were orthogonally manipulated to have either high, medium, or no visual noise added. The task was to respond to the picture and indicate whether it was a face or house. (B) There was a probabilistic relationship between the tones and pictures, which changed over time. Participants were blind to this ground truth structure, so any influence of the preceding tones on behavior was learned.

Here, we assessed the effects of propranolol, a common anxiolytic medication and β-adrenergic receptor antagonist,^37^ on sensory probabilistic associative learning. The task experimentally manipulated changes in the noisiness of a visual stimulus, the informativeness of a cue predicting its appearance (cue-outcome associations), and the volatility of these changing associations over time (Figure 1). ^29^^,^^38^^,^^39^ We employed a validated hierarchical Bayesian learning model that allowed us to characterize each participant’s learning “fingerprint”: how each subject learns about probabilistic relationships and volatility.^40^^,^^41^ Concurrent pupillometry served as a proxy for LC activity^31^, ^32^, ^33^ such that we could assess how global NA antagonism alters the relationship between trial-by-trial indices of prediction error and phasic LC function. We also collected cardiac and anxiety measures to verify the physiological effects of the propranolol manipulation and further investigate the proposed relationship between autonomic function and cognition.^42^

In a previous application of this task and model,^29^ we demonstrated behaviorally that adults with autism show *reduced* influence of expectations on reaction times (RTs). Computationally, this was linked to *increased* tendency to believe volatility is changing quickly and *heightened* encoding of trial-by-trial prediction errors in pupil size. On the basis of these results, we hypothesized a key role for raised noradrenergic function in altering the balance toward sensory inputs, and away from prior expectations, in autism.^3^^,^^29^ Therefore, in the current study we predicted that by blocking the action of NA (NA−), we would observe the opposite pattern of results: an *enhanced* effect of expectations on behavior, a *reduction* in volatility-linked learning, and a *diminished* encoding of trial-by-trial prediction errors on pupil size. This would provide direct evidence for the role of NA in learning under conditions of sensory uncertainty.

## Results

We employed a double-blind, placebo-controlled design in 40 healthy volunteers to test the effects of propranolol administration on a probabilistic associative learning (PAL) task, which quantifies the impact of learned expectations on RTs under conditions of sensory noise^29^^,^^38^^,^^39^^,^^43^ (see STAR Methods and Figure 1).

### Physiological Effects of Propranolol

**Figure 2:**
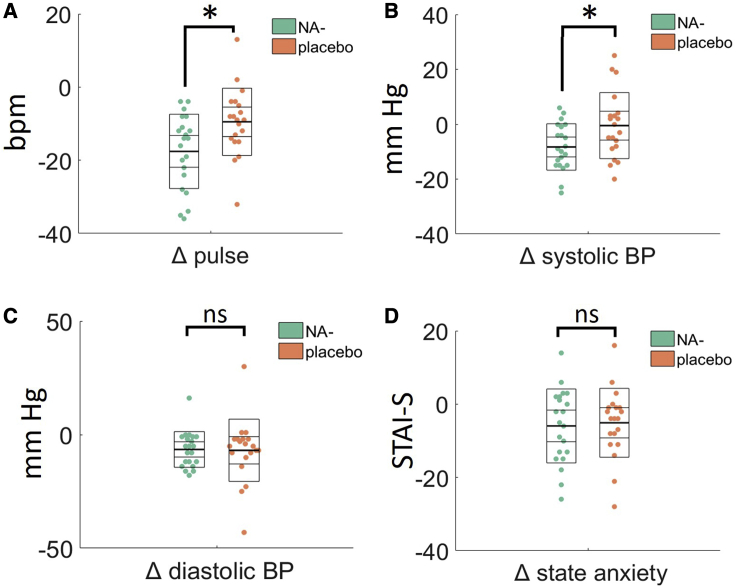
The Physiological and Mood Effects of Propranolol (NA−) Administration Plots show the change (Δ) in each measure from baseline (pre-drug administration) and 1-h post-drug administration. Propranolol (A) reduces heart rate and (B) reduces systolic blood pressure more than placebo but has no effect on (C) diastolic blood pressure or (E) state anxiety. Change in state anxiety (Δ) measured at baseline (before drug administration) an at the end of the testing session. Each data point represents an individual participant in the study. Thick middle line shows the mean, inner box shows the 95% standard error of the mean (SEM), and outer box shows the standard deviation. BP, blood pressure; Bpm, beats per minute; STAI-S, the state anxiety scale from the State-Trait Anxiety Inventory. ^∗^ denotes statistically significant difference between propranolol (NA−) and placebo groups. ns, not significant.

First we compared cardiac measures at baseline (before drug administration) and immediately before the tasks started (∼1 h post drug administration) to assess the acute effects of propranolol. There was a significant main effect of time point on pulse rate (F(1,38) = 80.96, p < 0.001, η_p_^2^ = 0.681), indicating that pulse reduced in all participants after 1 h in the experimental setting. However, a significant time × group interaction (F(1,38) = 10.35, p = 0.003, η_p_^2^ = 0.214) reflected the fact that pulse rate reduced significantly more in the propranolol group relative to the placebo group (Figure 2A). This was the same for systolic blood pressure (BP), which reduced overall (F(1,38) = 6.02, p = 0.02, η_p_^2^ = 0.137) but significantly more in the propranolol group (F(1,38) = 7.77, p = 0.008, η_p_^2^ = 0.170; Figure 2B). Diastolic BP reduced in all participants (F(1,38) = 13.22, p = 0.001, η_p_^2^ = 0.258), but there was no interaction with group (F < 1; Figure 2C). Finally, we compared state anxiety at baseline (before drug administration) and at the end of the testing session (∼2 h post drug administration). State anxiety reduced in all participants (F(1,38) = 13.69, p = 0.001, η_p_^2^ = 0.265), but this change in anxiety scores did not differ between the placebo and propranolol groups (F < 1; Figure 2D).

### Behavioral Results

We examined behavioral responses where expected (E) and unexpected (UE) trials were categorized according to the ground truth. RTs were submitted to a 2 × 3 mixed ANOVA with within-subjects factors of expectedness (E, UE), noise (high, medium, no), and a between participants factor of drug group (propranolol, placebo).

There was no main effect of group (F(1,38) = 0.154, p = 0.697, η_p_^2^ = 0.004) demonstrating that mean RT did not change as a function of taking propranolol. This rules out simple explanations (e.g., drowsiness) for any further group differences.

There was a significant linear effect of expectation (F(1,38) = 14.63, p < 0.001, η_p_^2^ = 0.278) and also noise (F(1,38) = 91.45, p < 0.001, η_p_^2^ = 0.706). This indicates that RT slows down as outcome images become both less expected and noisier. Consistent with the fact that expectations often exert greatest influence on behavior when sensory inputs are uncertain, there was a significant linear expectation × noise interaction (F(1,38) = 17.73, p = < 0.001, η_p_^2^ = 0.318), such that the RT difference between E and UE trials was most pronounced for the high noise stimuli.

This effect was qualified by a significant linear expectation × noise × drug group interaction (F(1,38) = 4.60, p = 0.038, η_p_^2^ = 0.11), which indicates that the balance between expectations and noise is altered by NA antagonism. All other two-way interactions with group were not significant (p > 0.17).

**Figure 3:**
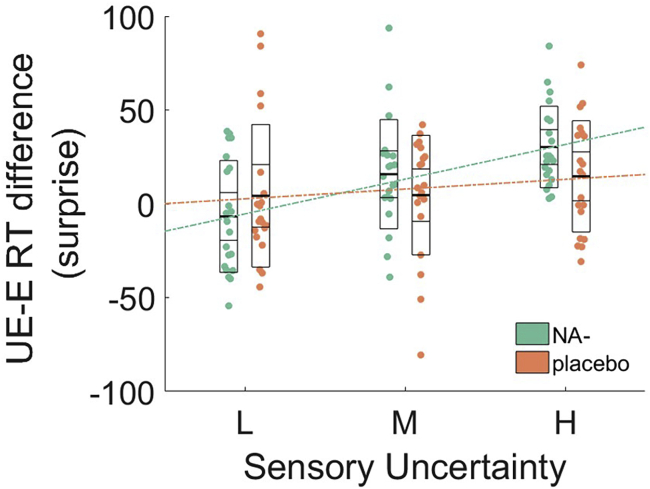
The Behavioral Effects of Propranolol (NA−) Administration Plot shows the difference in RT between unexpected (UE) and expected (E) trials (i.e., surprise) as a function of stimulus noise (sensory uncertainty). As noise increases from no/low (L), medium (M), to high (H), expectations exert more influence on behavior (i.e., RTs for unexpected stimuli increase), and this interaction is stronger in the propranolol group. L, low; M, medium; H, high; referring to low, medium, and high visual noise on the images. Each data point represents an individual participant. Thick middle line shows the mean, inner box shows the 95% SEM, and outer box shows the standard deviation. Dotted lines show linear fits.

Specifically, in the placebo group only, there are linear main effects of expectation (F(1, 18) = 4.90, p = 0.40, η_p_^2^ = 0.214) and noise (F(1, 18) = 39.18, p < 0.001, η_p_^2^ = 0.685), but the expectation × noise interaction is not significant (F(1, 18) = 1.5, p = 0.24, η_p_^2^ = 0.077). This replicates the results of our previous study employing this task.^29^ However, the expectation × noise interaction is significant under NA antagonism (F(1,20) = 31.09, p < 0.001, η_p_^2^ = 0.609), in addition to the two main effects (expectation: F(1, 20) = 10.50, p = 0.004, η_p_^2^ = 0.344; noise: F(1, 20) = 54.36, p < 0.001, η_p_^2^ = 0.731). In other words, the linear effect of expectations on noise is enhanced under propranolol (i.e., steeper slope; Figure 3).

### Computational Results

**Figure 4:**
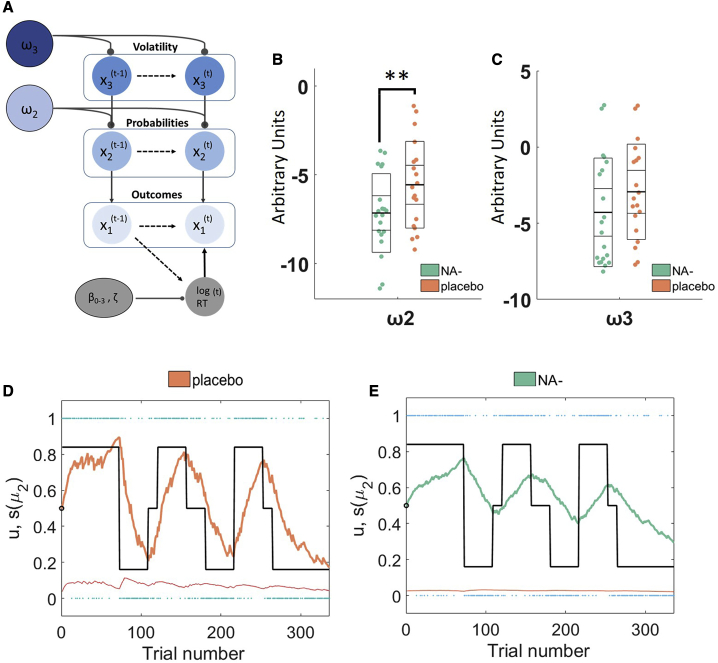
Computational Model Details and Results (A) A schematic of the HGF based on trial-by-trial logRT, the model estimates participants learning about probabilities $\left(\omega_{2}\right)$ and volatility $\left(\omega_{3}\right)$. See text for more details. (B and C) The effects of propranolol on $\omega_{2}$ and $\omega_{3}$ can be seen in (B) and (C), respectively. Propranolol significantly reduces probabilistic learning $\left(\omega_{2}\right)$ and moderately reduces beliefs about volatility $\left(\omega_{3}\right)$. Model parameter values are in arbitrary units, with higher values indicative of higher learning rates and volatility estimates, respectively. Data points represent individual participants. Thick middle line shows the mean, inner box shows the 95% SEM, and outer box shows the standard deviation.^∗∗^ denotes p < 0.05 (two-tailed). (D and E) Bayesian parameter average trajectories showing the effects of (D) placebo, relative to (E) propranolol on posterior beliefs about stimulus outcome μ_2_ across the experiment. Under propranolol (green) the Bayesian average participant learns the cue-outcome contingencies less readily and updates less in the face of stimuli that violate expectations. Thin red line shows the average estimated dynamic learning rate. Blue dots show the actual outcomes (u) encountered on each trial. Thick black lines show the “ground truth” changing P(image|tone). For exploratory analyses of group differences in the learning trajectories at the individual participant level, please see Figure S2.

To ensure that the chosen model (HGF; Figure 4A) performs well to describe the behavior of our participants, we fit three alternative learning models to the data and compared them with random-effects Bayesian model selection (BMS). Relative to simple reinforcement learning models with fixed (RW)^44^ and dynamic (SK1)^45^ learning rates and an alternative HGF where we omitted the influence of sensory noise (HGF_alt), the HGF was the best model for explaining the data by a considerable margin (see STAR Methods and Figure S1A).

The winning HGF model underwent subsequent validation steps including data simulations and demonstrations of parameter recovery (see STAR Methods and Figures S1B–S1D).

#### Differences in Model Parameters

First, we assessed whether there were group differences in the perceptual model parameters that capture individual differences in learning. This tests our *a priori* hypothesis that propranolol would slow learning. A binary logistic regression model predicting drug group with $\omega_{2}$ and $\omega_{3}$ as predictors was significant ($X^{2}$ = 6.85, df = 2, r^2^ = 0.22, p = 0.032), and $\omega_{2}$ significantly differed across groups (β = 0.36, p = 0.04 [two-tailed]; p = 0.02 [one-tailed], bootstrapped p = 0.011 [two-tailed], bootstrapped confidence interval [CI] [0.08–1.01]; Figure 4B). However, $\omega_{3}$ only approached, but did not reach, significance at a one-tailed level (β = 0.17, p = 0.14 [two-tailed], p = 0.07 [one-tailed], bootstrapped p = 0.15 [two-tailed], bootstrapped CI [−0.059–612]; Figure 4C). These results indicate that propranolol slows learning about cue-outcome contingencies and may produce a modest reduction in estimated volatility. Plotting the Bayesian parameter average posterior expectation of stimulus outcome for each group indicates that (consistent with a lower $\;\omega_{2}$), under propranolol, participants are less able to track the changing probabilities throughout the task (Figures 4D and 4E).

A binary logistic regression model predicting group status from the beta weights of the linear response model (b_0–3_, z) was not significant ($X^{2}$ = 3.89, df = 5, r^2^ = 0.12, p = 0.56), and there were no significant predictors (p > 0.38) indicating that propranolol did not broadly affect the mapping from the perceptual model to motor behavior.

#### Model-Based Pupillometry

In fitting the parameters of the perceptual model, the HGF gives rise to trial-by-trial estimates of prediction errors ($\varepsilon_{2,\;}$ $\varepsilon_{3}$) and volatility $\left(\mu_{3}\right)$, which reflect, for each participant, their personal learning process. We linked these trajectories to trial-by-trial changes in pupil size, a proxy measure for central noradrenergic function.^31^, ^32^, ^33^ We assessed the effects of NA antagonism on the encoding of “low-level” precision-weighted prediction errors about stimulus outcomes $\left(\varepsilon_{2}\right)$, “high-level” precision-weighted prediction errors about cue-outcome contingencies $\left(\varepsilon_{3}\right)$, and phasic volatility $\left(\mu_{3}\right)$.

**Figure 5:**
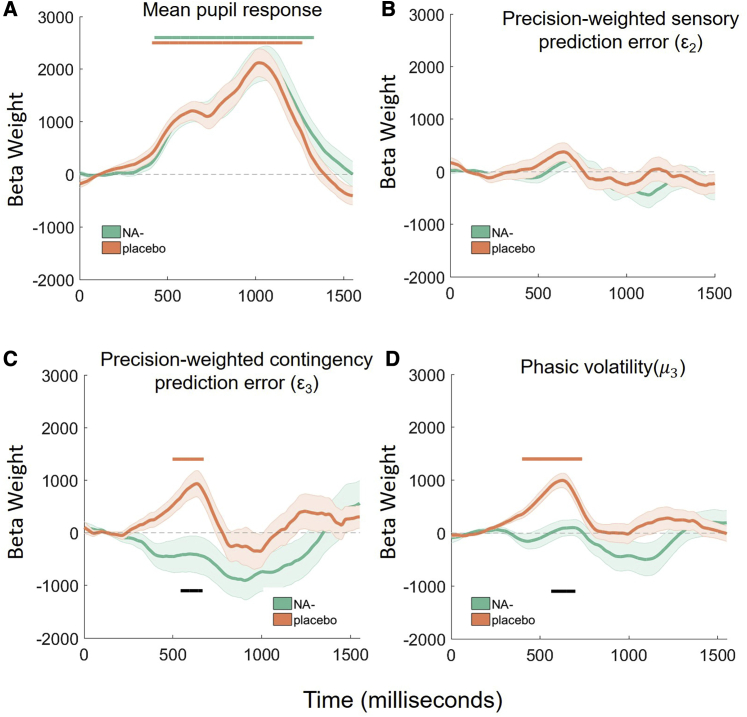
The Effects of Propranolol on Pupil Size Measurements Propranolol (A) does not change average stimulus-evoked pupil responses or (B) the phasic (trial-by-trial) relationship between pupil size and precision-weighted prediction errors about stimulus outcome. However, propranolol attenuates the relationship between pupil size and (C) precision-weighted prediction errors about contingencies and (D) volatility. In all panels, the orange solid bars indicate significant clusters when the pupil response differed from zero in the placebo group, green solid horizontal lines indicate the same for the propranolol group, and the black solid horizontal line shows time clusters where the propranolol group significantly differed from the placebo group (2,000 permutations: cluster *α* = 0.05). In all panels shaded error bars represent the SEM of the beta estimates across individual participants. In all pupil analyses, this regression approach controlled for other explanatory and nuisance variables on a trial-by-trial basis (see main text for more details).

There were no group differences in mean pupil size across all trials (Figure 5A), indicating that propranolol did not globally affect pupil responses. Consistent with previous studies,^29^ low-level precision-weighted prediction errors $\left(\varepsilon_{2}\right)$ were not encoded by pupil size in either group (Figure 5B). In the placebo group, both high-level precision-weighted prediction errors $\left(\varepsilon_{3}\right)$ and phasic volatility $\left(\mu_{3}\right)$ were encoded in pupil size, such that when estimates of prediction error or volatility were high, pupil size increased (Figures 5C and 5D). However, under propranolol, the relationship between $\varepsilon_{3}$ and $\mu_{3}$ with pupil size was significantly attenuated (Figures 5C and 5D; black lines). This suggests that under global tonic noradrenergic blockade of β-adrenoreceptors, the phasic functions of the LC are diminished.

#### Predicting Learning from Anxiety and Cardiac Measures

To assess the relationship between our computational estimates of learning under uncertainty and baseline cardiac and anxiety measures, we conducted two multiple linear regression models predicting $\omega_{2}$ and $\omega_{3}$, respectively. The predictors of interest were baseline systolic and diastolic BP, pulse rate, state and trait anxiety, group, and all interactions between group and these other predictor variables. Owing to multicollinearity among the predictors (variance inflation factors > 10), we employed stepwise methods to eliminate redundant predictors from the model and improve model fit (Δ Bayesian information criterion; BIC).

**Figure 6:**
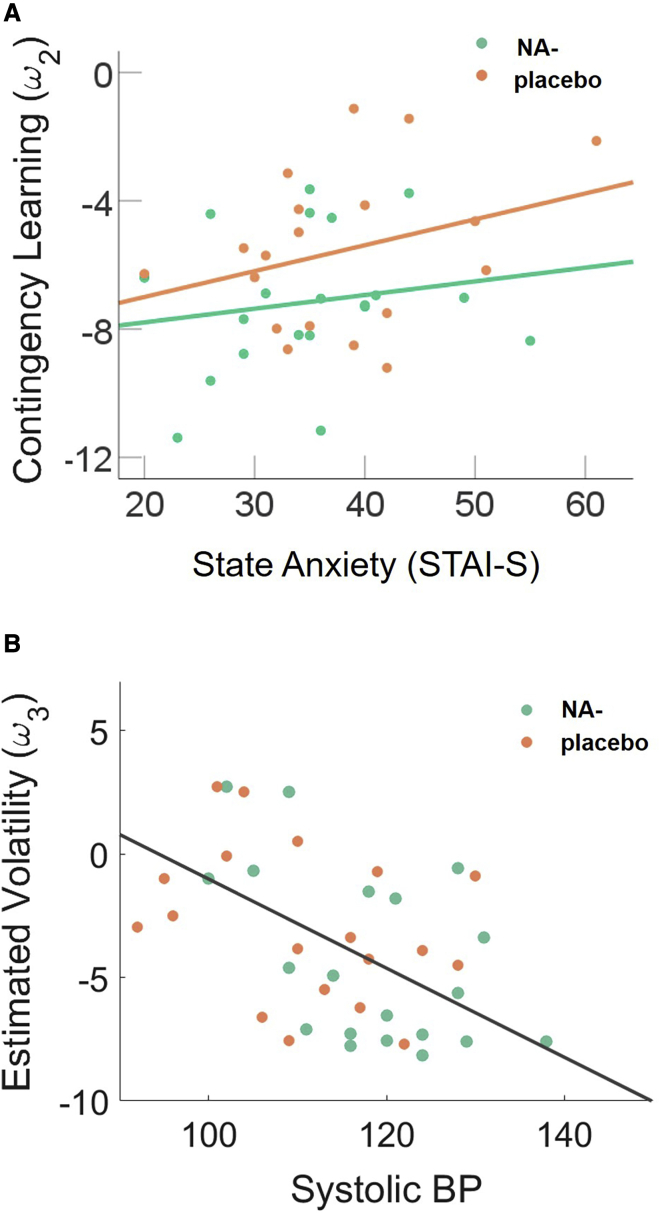
The Relationship between Learning under Uncertainty ($\omega_{2}$, $\omega_{3}$) and Anxiety/Cardiac Measures (A) Linear relationship between state anxiety and contingency learning, i.e., faster learning in high state anxious individuals, is attenuated by propranolol (NA−). (B) In both groups, baseline systolic blood pressure (mm Hg) predicts participants’ estimate of volatility in the task.

The resulting model predicting $\omega_{2}$ was significant (F(1, 38) = 7.34, p = 0.012, R^2^ = 0.166), and drug group interacted with state anxiety in predicting $\omega_{2}$ (β = 0.40, t = 2.71, p = 0.01; Figure 6A). This indicates that the slope of the line relating contingency learning to baseline state anxiety differs across drug groups. Specifically, for high state anxious individuals contingency learning rates were generally higher, but this effect was diminished under propranolol. The final model predicting $\omega_{3}$ was significant (F(1, 38) = 9.74, p = 0.003, R^2^ = 0.21) and baseline systolic BP was a significant predictor of $\omega_{3}$ (β = −.46, t = −.312, p = 0.003; Figure 6B). This suggests that individuals with higher baseline systolic BP tend toward lower estimates of $\omega_{3}$ during probabilistic learning (i.e., they estimate the environment as less volatile).

We corroborated these results when performing these analyses using regularized Ridge regression,^46^ which penalizes, rather than eliminates, redundant predictors (the group-by-state-anxiety interaction was the only significant predictor of $\omega_{2}$ (t = 2.43, p = 0.0151); baseline systolic BP was the only significant predictor of $\omega_{3}$ (t = 3.161, p = 0.00157)).

## Discussion

Learning what to expect in a changing world is central to adaptive behavior, and requires coordination across complex learning dynamics, central sensory systems, and peripheral physiological states. Here, we administered propranolol during a probabilistic learning task that orthogonally varied sensory noise, probabilistic uncertainty, and volatility in combination with computational and physiological measures. We discovered that β-adrenergic receptor blockade slowed contingency learning, especially in high state anxious individuals, and reduced the effects of prediction errors and volatility on pupil size. Across all participants, cardiac measures also predicted volatility updates.

Previous studies have reported that decision time increases linearly with sensory uncertainty (i.e., noise^29^^,^^39^^,^^47^ and unexpectedness^29^^,^^39^^,^^43^^,^^48^). Indeed, these two factors interact such that our prior expectations often exert the greatest influence on behavior when sensory inputs are uncertain^29^^,^^39^^,^^49^). Here, we showed that this interaction is augmented under noradrenergic blockade (Figure 3). This finding occurs in the absence of any main effect of propranolol on reaction times, indicating that our drug manipulation did not have gross effects on motor function or alertness. This is consistent with our recent report of diminished effects of expectations on behavior in autism.^29^ A reduced influence of prior expectations during perception might underlie the sensory overload symptoms of autism;^1^^,^^2^^,^^4^ additionally, both anxiety and PTSD are described as states in which people are hyper-vigilant to potential environmental threats (i.e., disproportionately sensitive to sensory inputs^11^^,^^13^^,^^50^^,^^51^). Consistent with these accounts, our findings indicate that the anxiolytic properties of propranolol might act by increasing the strength or confidence of one’s prior beliefs at the expense of sensory drive. Indeed, a recent study found that acute anxiety shifts neural dynamics toward feedforward processing of sensory inputs, with “top-down” feedback connectivity restored after taking anxiolytic medication.^11^ These findings, in line with our observation of faster sensory learning in high anxious individuals (Figure 6A), support sensory hypervigilance accounts of anxiety.^11^

The hierarchical Bayesian learning model that we employed allowed us to interrogate the effects of propranolol on the learning mechanisms that give rise to our prior expectations. This novel modeling approach builds on decades of research on fear learning in anxiety^52^ but extends this work to non-aversive sensory learning. However, the relationship between anxious states and different domains of learning is still not clear. Our finding that propranolol slows sensory learning is consistent with studies that have found learning in other domains (e.g. negative outcomes^53^ and safety^54^) is heightened in anxiety. However, recent work suggests that inducing anxiety in the lab might slow reward-based learning.^55^ We are keen to stress that in our study the participants were not clinically anxious, there was no manipulation of state anxiety and, accordingly, propranolol did not significantly reduce self-report measures of anxiety. More work is needed to bridge across the different domains of learning and anxious states to fully understand the anxiolytic properties of propranolol.

Our computational results (Figure 4B) suggest that propranolol slows down learning about cue-outcome contingencies $\left(\omega_{2}\right)$. This is consistent with work in non-human animals showing that NA blockade impairs associative and also reversal learning.^24^ Slower contingency learning means that individuals will weight their prior expectations to a greater extent and be less likely to update their beliefs in the face of new information (as can be visualized in Figure 4E). In our model, this update is proportional to the noise on the stimulus, with smaller updates when the sensory inputs are less certain. Accordingly, the simple behavioral effect of expectations on reaction times is enhanced under propranolol and most pronounced for high noise stimuli. Recent theoretical accounts have made efforts to highlight the relationships between model uncertainty (i.e., the relationships between sensory states) and sensory uncertainty (i.e., noise) and propose that common gain control mechanisms might underlie the computation of both.^56^, ^57^, ^58^ Our results demonstrate such a mechanistic link and, in doing so, highlight the intimate commonalities between perception and learning under uncertainty.

Relying more on prior expectations, by updating less in response to unexpected stimuli, is optimal in a stable environment. In a volatile environment, one ought to update their beliefs readily to learn quickly about the changing state of the world.^26^^,^^27^^,^^29^ Seminal theoretical accounts posit that NA plays a key role in signaling volatility (cf. unexpected uncertainty^15^^,^^16^). We previously found increased volatility updating $\left(\omega_{3}\right)$ in autism, and pupillometric measures indicated that this could be a consequence of a hyper-noradrenergic state.^29^ Futheremore, recent work has shown that enhancing the action of the catecholamines, dopamine, and noradrenaline increases learning under volatile conditions.^59^ We therefore predicted *a priori* that blocking the action of NA would reduce volatility-linked learning. Our data and simulations indicate that noradrenergic blockade might produce a moderate reduction in $\omega_{3}$ (Figure 4C; Figures S1C and S1D). However, this result should be interpreted conservatively since it only approached significance with a one-tailed statistical test. It is interesting to note that a recent study tested the effect of α-adrenergic blockade on motor learning and found a moderate increase in estimated volatility.^36^ Propranolol is a potent β-adrenergic antagonist, which also weakly stimulates α_1_-adrenoceptors.^60^ It is therefore possible that this effect on α-receptors might in fact be driving the modest reduction in volatility in our present study. Alternatively, one intriguing possibility is that α- and β-adrenergic receptors have opposing effects on volatility, with one acting to stabilize and the other destabilize the estimate of the rate with which volatility changes. Future studies could investigate this possibility by contrasting the effects of selective α- versus β-adrenergic antagonists using the same task and model.

Prior work has shown that dynamic trial-by-trial changes in volatility^27^^,^^29^ and “high-level” contingency prediction errors^29^ are encoded in pupil size. These studies in humans, building on work in rodents^22^^,^^61^ and primates,^32^ provide evidence for noradrenergic involvement in signaling unexpected changes. Here, we show that the phasic trial-by-trial relationship between both contingency prediction errors and volatility and pupil size is diminished under global β-adrenergic receptor blockade (Figure 5). These findings add further empirical support to the suggestion that pupil diameter serves as a useful peripheral readout of central NA function in humans.

Finally, we show for the first time to our knowledge that cardiovascular function is linked to learning about volatility under conditions of uncertainty. Specifically, low baseline systolic BP is predictive of higher subsequent beliefs about volatility in the task (Figure 6A). We note that in the context of this nonclinical sample higher estimates of volatility (in this volatile task environment) could be regarded as adaptive, and this is predicted by lower (healthier) baseline BP. Alterations in the ability to learn about volatility have been recently implicated in a number of clinical conditions, including autism,^29^^,^^62^ anxiety,^27^^,^^28^ and psychosis,^30^ which also share common cardiac pathology such as increased risk for cardiometabolic diseases.^63^ At a recent summit for interoception, key figures in the research community set out a roadmap for understanding body-brain interactions in cognition and mental health.^64^ Mechanistically, directional communication between the brain and the internal organs is mediated via the vagus nerve, which coordinates the HPA axis to produce adaptive stress responses. Visceral inputs from the vagus nerve project to the LC, raphe nucleus, and other cortical regions to impact on cognition, neurotransmission, and brain connectivity in humans.^65^ Situated within a rapidly growing number of theoretical perspectives on embodied cognition,^66^^,^^67^ our empirical results suggest that global cardiac state might play a role in setting the boundary conditions for the rate that relationships between sensory events is estimated to change. This is interesting in the context of recent work showing that vagal nerve stimulation can reduce learning rates during reinforcement learning^68^ and that blood pressure, ^69^ and other aspects of cardiac function, impacts on cognition.^70^, ^71^, ^72^

In summary, our results provide a novel insight into the behavioral, computational, and physiological effects of NA on learning under conditions of uncertainty. Our findings underscore the relationship between the cognitive and autonomic effects of anxiety and offer computational insights into the possible mechanisms underlying therapeutic effects of beta blockers.

## STAR★Methods

### Key Resources Table

**Table:** 

REAGENT or RESOURCE	SOURCE	IDENTIFIER
**Software and Algorithms**		

MATLAB	https://www.mathworks.com/	RRID:SCR_001622
SPSS	https://www.ibm.com/uk-en/products/spss-statistics	RRID:SCR_002865
RStudio	https://www.rstudio.com/	RRID:SCR_000432
HGF Toolbox	TAPAS https://www.tnu.ethz.ch/en/software/tapas	N/A
Mass Univariate ERP Toolbox	https://openwetware.org/wiki/Mass_Univariate_ERP_Toolbox	N/A

### Resource Availability

#### Lead Contact

Further information and requests for resources directed to the Lead Contact, Dr Rebecca Lawson (rl337@cam.ac.uk).

#### Materials Availability

This study did not generate any new unique reagents

#### Data and Code Availability

Summary data and code generated during this study are available at: [https://github.com/BeckyLawson/Propranolol].

### Experimental Model and Subject Details

Forty adult human community volunteers (21 females) were tested using a double-blind, placebo-controlled design. All participants had normal or corrected-to-normal vision and reported no prior history of neuropsychiatric illness, or previous conditions or medication that contraindicate the administration of 40mg propranolol (cardiovascular disease, lung/ kidney/liver disease, substance use, pregnancy).

The study was approved by the UCL Ethics Committee (#1338/007).

### Method Details

#### Drug Manipulation

Participants were randomized to one of two experimental conditions (propranolol, placebo). Contraindications were checked and drugs were administered by a qualified clinician (AL). The experimenter was blind to drug group and unblinding only occurred after the study was completed. There were no group differences in sex, age, anxiety, or cardiac measures (Table 1). One participant (propranolol group) was excluded from the computational analysis owing to a high proportion of error responses, which could affect the Bayesian modeling.^29^

**Table 1:** Participant Characteristics

	Propranolol (n= 21)	Placebo (n = 19)	Between-Group Difference
Mean age (SD)	23.47 (6.00)	26.58 (8.36)	t(38) = 1.36, p = 0.18
Diastolic blood pressure	75.62 (10.44)	74.63 (13.60)	t(38) = 0.26, p = 0.80
Systolic blood pressure	118.00 (9.93)	111.16 (11.12)	t(38) = 2.1, p = 0.05
Heart rate	79.29 (18.21)	83.84 (12.19)	t(38) = 0.92, p = 0.36
Baseline state anxiety	35.19 (8.36)	37.84 (9.31)	t(38) = 0.95, p = 0.35

#### General Procedure

Each participant attended one experimental session during which they received a single oral dose of either 40mg propranolol or a placebo (vitamin D). We selected doses that were in line with previous studies showing clear behavioral and neurophysiological effects of noradrenaline blockade.^73^^,^^74^ On arrival participants gave written informed consent and self-report measures of state and trait anxiety.^75^ A baseline heart rate and blood pressure measurement was taken using an *Omron M7 Intellisense* automatic blood pressure monitor. After drug administration, 60 min passed to allow propranolol to reach its peak levels.^76^ A second round of cardiac measurements were taken at this time, after which participants started the cognitive testing. A third round of cardiac measures was taken at the end of the experimental session, approximately 50 min after the last pre-task measures.

#### Task & Stimuli

We used a probabilistic associative learning (PAL) task to test the impact of learned expectations on reaction times (RT) under conditions of sensory noise.^29^^,^^38^^,^^39^^,^^43^

The task consisted of 336 trials split over three short blocks lasting ∼8 min each (see Figure 1). On each trial, participants performed binary classification of images which were either faces or houses. All images were luminance matched across conditions using the SHINE toolbox.^77^ Each image was preceded by a tone (440Hz or 660Hz pure tone) that was either highly-, non- or weakly-predictive of a given outcome (face or house; Figure 1A), and these associations themselves changed unpredictably across time (environmental volatility, Figure 1B). Trials were classified according to whether the image was expected (P(image|tone) = 0.84), neutral (P(image|tone) = 0.5) or unexpected (P(image|tone) = 0.16). To maximize the influence of expectation on behavior, visual uncertainty was orthogonally manipulated such that images had either high, medium, or no visual noise added (Figure 1A). High, medium and no noise stimuli were equally distributed across the expected, neutral, and unexpected trials. The noise (high, medium, no) and expectation (highly and weakly-predictive) conditions in this task allowed us to test for the main effect of expectation, main effect of noise, and the interaction between these two factors.

Participants were instructed to respond to the images, indicating on each trial whether they saw a face or a house. Participants were not explicitly instructed about the probabilistic relationship between the tones and the outcomes. Therefore, any effect of the preceding tones (i.e., expectations) on reaction time, were a result of participants implicitly picking up on the probabilistic structure of the task.

We note that in the design we have adopted here, as in,^28^^,^^29^^,^^36^^,^^38^^,^^39^ the unexpected changes in contingencies throughout the task means that volatility has to be estimated and learned about continuously. This is in contrast to designs where volatility only changes once, between blocks of stable and volatile contingencies.^26^^,^^27^ Accordingly, a three level HGF model which includes, learning about volatility itself, demonstrably fits the data better than simple R-W models (see Figure S1A).

#### Pupillometry

Pupil size was measured during the task with an infrared eye tracker (SR-Research Eyelink 1000) tracking at 1000 Hz. Chin and forehead were stabilized using a table mounted head rest. Calibration of the eye tracker was unsuccessful in three participants, one in the placebo group and two in the propranolol group.

### Quantification and Statistical Analysis

#### Behavioral analysis

All statistical analysis of behavioral data was performed in MATLAB (Mathworks, Ltd.), IBM SPSS Statistics (version 26) and R Studio, Version 1.2.1091. To maximize trial numbers per condition we collapsed across face/house trials and submitted RTs to a mixed ANOVA with within-subject factors of expectedness (unexpected (UE), expected (E)) and stimulus noise (high (H), medium (M) and no (N)), and a between-subjects factor of group. Since RT increases linearly with both surprise and stimulus noise^29^^,^^39^ we tested for the linear main effects of both and their interaction with drug condition. All statistical tests are reported at two-tailed level of significance unless explicitly stated.

#### Computational analysis

Model-agnostic behavioral analyses assume that each participant has learned the ground truth of the experiment to the same extent, i.e., trials experienced as ‘unexpected’ to one participant ought to be ‘unexpected’ to another. However, humans face a complex set of learning problems when trying to build expectations about the world, and the optimal way to update one’s behavior under uncertainty is to use Bayesian inference. Therefore, we employed a participant-specific Bayesian learning model (the Hierarchical Gaussian Filter (HGF)^40^^,^^78^) to track the role of uncertainty on behavior. The input to the model is trial-by-trial log RT, making the HGF employed in this study (and in prior work employing conceptually similar tasks^29^^,^^38^^,^^48^) a model of behavior in the simplest sense. The version of the HGF applied here (Figure 4A) has been used previously to examine both cultural and clinical differences in expectation learning,^29^^,^^39^ therefore succinct model details are provided below to avoid unnecessary repetition.

##### The perceptual model

The HGF’s perceptual model tracks a participant’s beliefs about the task structure: the trial wise stimulus outcomes at level 1 $\left(x_{1}\right)$, the probabilistic relationship between the tone and the outcome at level 2 $\left(x_{2}\right)$, and the volatility of these relationships at level 3 $\left(x_{3}\right)$. Two participant-specific perceptual parameters are estimated, $\omega_{2}$ and $\omega_{3}$, and allow for individual differences in approximate Bayes-optimal learning at each level:

$$x_{1}^{\left(t\right)}∼\text{Bernoulli}\left(s\left(x_{2}^{\left(t\right)}\right)\right),$$

$$x_{2}^{\left(t\right)}∼N\left(x_{2}^{\left(t-1\right)},\exp\left(x_{3}^{\left(t\right)}+\omega_{2}\right)\right),$$

$$x_{3}^{\left(t\right)}∼N\left(x_{3}^{\left(t-1\right)},\exp\left(\omega_{3}\right)\right),$$

$\omega_{2}$ captures a tonic evolution (or learning) rate at which probabilistic relationships are estimated to change. $\omega_{3}$ determines the rate at which estimates of phasic volatility are updated with higher values indicating a belief that volatility is changing quickly.

At any level i of the hierarchy, these two parameters give rise to trial-by-trial trajectories of belief updates (i.e., posterior mean $\mu_{i}^{\left(t\right)}$ of the state *x_i_*) that are proportional to the precision-weighted prediction error (PE) $\varepsilon_{i}^{\left(t\right)}$. This weighted PE is the product of the PE $\delta_{i-1}^{\left(t\right)}$ from the level below and a precision ratio $\left({\hat{\pi }}_{i-1}^{\left(t\right)}/\pi_{i}^{\left(t\right)}\right)$, Where $\pi_{i}^{\left(t\right)}$ is the posterior precision (inverse variance) at the current level and ${\hat{\pi }}_{i-1}^{\left(t\right)}$ is the precision (inverse variance) of the prediction at level below. Accordingly, $\varepsilon_{i}^{\left(t\right)}$, is the precision weighted-prediction error at a given level i:

$$\varepsilon_{i}^{\left(t\right)}=\frac{{\hat{\pi }}_{i-1}^{\left(t\right)}}{\pi_{i}^{\left(t\right)}}\delta_{i-1}^{\left(t\right)}$$

##### Linear mapping to RTs

The response model captures the mapping from a participant’s trial-wise beliefs (arising from the perceptual model) onto behavioral responses (log RTs). It is a simple linear model of the form:

$$\log\;RT^{\left(t\right)}∼N\left(\beta_{0}+\beta_{1}\cdot {\text{surprise}}^{\left(t\right)}+\beta_{2}\cdot \varepsilon_{3}^{\left(t\right)}+\beta_{3}\cdot {\text{volatility}}^{\left(t\right)},\zeta \right),$$

Here, ${\text{surprise}}^{\left(t\right)}$ is the Shannon surprise associated with the outcome $x_{1}$, weighted by the stimulus noise on any given trial (t). $\varepsilon_{3}^{\left(t\right)}$ is the precision-weighted prediction error about cue-outcome contingencies at $x_{2}$ and ${\text{volatility}}^{\left(t\right)}$ is the exponential of the estimate phasic log-volatility at $x_{3}$. The betas represent predictors which may be reasonably hypothesized to affect log RT and be impacted by noradrenergic manipulation. These same predictors were recently employed in a computational pharmacological study examining the effects of α-receptor noradrenergic antagonism on motor sequence learning,^36^ and so we wanted to assess equivalent effects of β-adrenergic antagonism in the present study. See supplemental information for model comparison procedures.

##### Bayesian Parameter Averaging

In addition to frequentist statistical comparisons of the model parameters estimated for the individual participants in the placebo and propranolol groups, we also captured the effects on the learning process with Bayesian Parameter Averaging (BPA).^79^ BPA has been employed in prior studies^36^^,^^80^ to demonstrate the fixed effects average over the subject level parameters, weighted by their precision and taking into account the covariance between parameters at the individual subject level.^19^

#### Model validation

We compared the HGF to two simpler learning models, one (RW; Rescorla and Wagner 1972) in which a single learning rate parameter is estimated for each participant and another (SK1;^45^ with a dynamic learning rate that varies trial-to-trial but does not learn about volatility. As a third comparison model (HGF_alt) we fit the same perceptual model for the HGF but changed the linear mapping to responses such that the ${\text{surprise}}^{\left(t\right)}$ predictor did not contain an interaction with stimulus noise.

To disambiguate these alternative explanations (models) for the participants’ behavior, we used BMS, which evaluates the relative plausibility of competing models in terms of their log evidences while adjusting for the trade-off between accuracy (fit) and complexity.^81^ The three level HGF is shown to be the winning model (Figure S1A)

We also simulated 100 virtual agents using the mean parameters of the propranolol and placebo groups. Categorisation of these RTs according to trial type (E, UE) and stimulus noise (H, M, L) showed that the main behavioral effects can be recapitulated by the model (Figure S1B). Statistical analysis of this simulated data confirmed the same results as the real participant behavior, notably a significant linear main effect of stimulus noise (F(1,197) = 23.06, p < 0.001), and a linear noise ^∗^ drug group interaction (F(1,197) = 4.53, p = 0.035).

We inverted the parameters of the winning model using simulated data to determine if we could recover the parameters and the statistical differences reported between the groups in the main text. First, we simulated 200 datasets using mean parameters for the propranolol and placebo groups and fit the model to these data (Figure S1C). A binary logistic regression predicting group (propranolol, placebo) from the recovered $\omega_{2}$ and $\omega_{3}$ parameters, was significant overall ($X^{2}$ = 270.73, df = 2, r^2^ = 0.65, p < 0.001), with $\omega_{2}$ and $\omega_{3}$ both significantly lower in the propranolol group (b = 0.78, p < 0.001; b = 1.2, p < 0.001). Furthermore, to show that we can also recover parameters across the range of values estimated from real participant data we also simulated data using the parameters from the individual participant model fits reported in the main text, averaging across 20 simulations per participant (Figure S1D). For these parameters, the binary logistic regression predicting group approached significance ($X^{2}$ = 4.99, df = 2, r^2^ = 0.157, p = 0.08). In this model $\omega_{2}$ was significantly lower in the propranolol group (b = 0.55, p = 0.042), whereas $\omega_{3}$ was numerically, but not statistically lower in the propranolol group (b = 0.09, p = 0.59). Taken together, these analyses broadly recapitulate the primary findings reported in the main text, where $\omega_{2}$ is significantly reduced under propranolol but the reduction in $\omega_{3}$ is less reliable, not reaching significance at a 2-tailed level.

Finally, we examined the two-way mixed effects intra-class correlation coefficients (ICC) for the real $\omega_{2}$ and $\omega_{3}$ parameters estimated for each single subject against the mean of the recovered parameters for each participant. Our ICC analysis sought absolute agreement between measures, and so accounted for systematic differences.^82^ The ICC between the real and recovered values of $\omega_{2}$ was 0.860 (CI: 0.69-0.94; p < 0.001), and the ICC between the real and recovered values of $\omega_{3}$ was 0.870(CI: 0.75-0.93; p < 0.001). ICC coefficients of 0.81–.1.00 are considered ‘almost perfect’^83^, suggesting that the reliability of the parameter recovery for this model is encouraging.

#### Pupill**ometry analysis**

Statistical analyses of eye tracking data were performed in MATLAB (Mathworks, Ltd.) In line with previous studies, only trials in which 80% or more samples were successfully tracked were included in the analysis, blinks were treated with linear interpolation and the resulting pupil traces were low-pass filtered and smoothed following the conventions outlined in Jackson and Sirois.^84^ To explore phasic pupil responses for correct trials, traces were baseline corrected to the average response during the 200ms preceding the outcome image.

We conducted multiple regression analyses at every time point to examine the relationship between pupil size and:

1 ε_2_, the precision-weighted PE about visual stimulus outcome (that serves to update the estimate of visual stimulus probabilities)
2 ε_3_, the precision-weighted PE about cue-outcome contingency (that serves to update the estimate of log-volatility).
3 μ_3,_ phasic log-volatility at the third level.

The resultant timeseries of β weights (multiple regression conducted at every time point) provided estimates of when and how the computationally derived metrics of surprise were encoded in pupil size, e.g., positive β weights indicate that when prediction error was high, pupil size increased.

This approach has recently been used to assess differences in the relationship between prediction-errors, volatility and pupil size in autistic and anxious adults.^27^^,^^29^ The post-outcome period for each trial was sampled using 775 2ms time bins. Regression analyses were conducted for each post-stimulus time bin, with HGF estimates of precision-weighted prediction errors $\left(\varepsilon_{2\;,\;}\varepsilon_{3}\right)$ and phasic volatility (μ_3_) as regressors of interest, plus the ‘ground truth’ contrast of unexpected (1) minus expected (−1) trials, outcome image (0 = face, 1 = house), stimulus noise (high, med, no) and RT for each trial entered as control regressors. We note that the ground truth contrast of expected minus unexpected trials is the same for all participants and captures when they “ought” to have been surprised (assuming perfect learning), whereas the precision-weighted prediction errors capture, for each individual participant, the modeled estimate of when their expectations were violated and to what extent. This will align closely with the ground truth in some participants and less so in others. Accordingly, $\varepsilon_{3}$ and the ground truth are weakly (r = 0.1294), but significantly correlated (p < 0.001). We included both measures as predictors in these regressions to demonstrate the utility of the model-based approach.

At the group level, we then conducted t tests for the positive or negative effects of the regressors of interest, and the independent-samples difference between groups, corrected for multiple comparisons with a cluster-based permutation approach at 2,000 permutations (cluster α = 0.05).^85^ This approach protects against false positives across correlated measurements (i.e., maximizes temporal sensitivity).

## Supplemental Information

Document S1. Figures S1 and S2 and Supplemental References

Document S2. Article plus Supplemental Information

## References

[1] Friston K.J., Lawson R., Frith C.D. (2013). On hyperpriors and hypopriors: comment on Pellicano and Burr. Trends Cogn. Sci..

[2] Lawson R.P., Rees G., Friston K.J. (2014). An aberrant precision account of autism. Front. Hum. Neurosci..

[3] Palmer C.J., Lawson R.P., Hohwy J. (2017). Bayesian approaches to autism: Towards volatility, action, and behavior. Psychol. Bull..

[4] Pellicano E., Burr D. (2012). When the world becomes ‘too real’: a Bayesian explanation of autistic perception. Trends Cogn. Sci..

[5] Fletcher P.C., Frith C.D. (2009). Perceiving is believing: a Bayesian approach to explaining the positive symptoms of schizophrenia. Nat. Rev. Neurosci..

[6] Adams R.A., Stephan K.E., Brown H.R., Frith C.D., Friston K.J. (2013). The computational anatomy of psychosis. Front. Psychiatry.

[7] Vinckier F., Gaillard R., Palminteri S., Rigoux L., Salvador A., Fornito A., Adapa R., Krebs M.-O., Pessiglione M., Fletcher P.C. (2016). Confidence and psychosis: a neuro-computational account of contingency learning disruption by NMDA blockade. Mol. Psychiatry.

[8] Sterzer P., Adams R.A., Fletcher P., Frith C., Lawrie S.M., Muckli L. (2018). The Predictive Coding Account of Psychosis. Biological psychiatry.

[9] Paulus M.P., Stein M.B. (2006). An insular view of anxiety. Biol. Psychiatry.

[10] Clark J.E., Watson S., Friston K.J. (2018). What is mood? A computational perspective. Psychol. Med..

[11] Cornwell B.R., Garrido M.I., Overstreet C., Pine D.S., Grillon C. (2017). The unpredictive brain under threat: A neurocomputational account of anxious hypervigilance. Biol. Psychiatry.

[12] Wilkinson S., Dodgson G., Meares K. (2017). Predictive Processing and the Varieties of Psychological Trauma. Front. Psychol..

[13] Seriès P. (2019). Post-traumatic stress disorder as a disorder of prediction. Nat. Neurosci..

[14] Linson Adam, Friston Karl (2019). Reframing PTSD for computational psychiatry with the active inference framework. Cognitive Neuropsychiatry.

[15] Yu A., Dayan P. (2003). Expected and unexpected uncertainty: ACh and NE in the neocortex. Adv. Neural Inf. Process. Syst..

[16] Yu A.J., Dayan P. (2005). Uncertainty, neuromodulation, and attention. Neuron.

[17] Berridge C.W., Waterhouse B.D. (2003). The locus coeruleus-noradrenergic system: modulation of behavioral state and state-dependent cognitive processes. Brain Res. Brain Res. Rev..

[18] Lee S.-H., Dan Y. (2012). Neuromodulation of brain states. Neuron.

[19] Parr T., Rees G., Friston K.J. (2018). Computational neuropsychology and Bayesian inference. Front. Hum. Neurosci..

[20] Hasselmo M.E., Linster C., Patil M., Ma D., Cekic M. (1997). Noradrenergic suppression of synaptic transmission may influence cortical signal-to-noise ratio. J. Neurophysiol..

[21] Kobayashi M., Imamura K., Sugai T., Onoda N., Yamamoto M., Komai S., Watanabe Y. (2000). Selective suppression of horizontal propagation in rat visual cortex by norepinephrine. Eur. J. Neurosci..

[22] Martins A.R.O., Froemke R.C. (2015). Coordinated forms of noradrenergic plasticity in the locus coeruleus and primary auditory cortex. Nat. Neurosci..

[23] Glennon E., Carcea I., Martins A.R.O., Multani J., Shehu I., Svirsky M.A., Froemke R.C. (2018). Locus coeruleus activation accelerates perceptual learning. Brain Res..

[24] Ridley R.M., Haystead T.A.J., Baker H.F., Crow T.J. (1981). A new approach to the role of noradrenaline in learning: problem-solving in the marmoset after α-noradrenergic receptor blockade. Pharmacol. Biochem. Behav..

[25] Janitzky K., Lippert M.T., Engelhorn A., Tegtmeier J., Goldschmidt J., Heinze H.-J., Ohl F.W. (2015). Optogenetic silencing of locus coeruleus activity in mice impairs cognitive flexibility in an attentional set-shifting task. Front. Behav. Neurosci..

[26] Behrens T.E., Woolrich M.W., Walton M.E., Rushworth M.F. (2007). Learning the value of information in an uncertain world. Nat. Neurosci..

[27] Browning M., Behrens T.E., Jocham G., O’Reilly J.X., Bishop S.J. (2015). Anxious individuals have difficulty learning the causal statistics of aversive environments. Nat. Neurosci..

[28] de Berker A.O., Rutledge R.B., Mathys C., Marshall L., Cross G.F., Dolan R.J., Bestmann S. (2016). Computations of uncertainty mediate acute stress responses in humans. Nat. Commun..

[29] Lawson R.P., Mathys C., Rees G. (2017). Adults with autism overestimate the volatility of the sensory environment. Nat. Neurosci..

[30] Powers A.R., Mathys C., Corlett P.R. (2017). Pavlovian conditioning-induced hallucinations result from overweighting of perceptual priors. Science.

[31] Murphy P.R., O’Connell R.G., O’Sullivan M., Robertson I.H., Balsters J.H. (2014). Pupil diameter covaries with BOLD activity in human locus coeruleus. Hum. Brain Mapp..

[32] Joshi S., Li Y., Kalwani R.M., Gold J.I. (2016). Relationships between pupil diameter and neuronal activity in the locus coeruleus, colliculi, and cingulate cortex. Neuron.

[33] Larsen R.S., Waters J. (2018). Neuromodulatory Correlates of Pupil Dilation. Front. Neural Circuits.

[34] Nassar M.R., Rumsey K.M., Wilson R.C., Parikh K., Heasly B., Gold J.I. (2012). Rational regulation of learning dynamics by pupil-linked arousal systems. Nat. Neurosci..

[35] Jepma M., Murphy P.R., Nassar M.R., Rangel-Gomez M., Meeter M., Nieuwenhuis S. (2016). Catecholaminergic Regulation of Learning Rate in a Dynamic Environment. PLoS Comput. Biol..

[36] Marshall L., Mathys C., Ruge D., de Berker A.O., Dayan P., Stephan K.E., Bestmann S. (2016). Pharmacological Fingerprints of Contextual Uncertainty. PLoS Biol..

[37] Brudkowska Ż., Tomczyk M., Jusiak K., Karakuła-Juchnowicz H., Rudnicka-Drożak E. (2018). The role of beta-adrenolytic drugs in treating anxiety disorders. Current Problems of Psychiatry.

[38] Iglesias S., Mathys C., Brodersen K.H., Kasper L., Piccirelli M., den Ouden H.E., Stephan K.E. (2013). Hierarchical prediction errors in midbrain and basal forebrain during sensory learning. Neuron.

[39] Wright N.D., Grohn J., Song C., Rees G., Lawson R.P. (2018). Cultural effects on computational metrics of spatial and temporal context. Sci. Rep..

[40] Mathys C., Daunizeau J., Friston K.J., Stephan K.E. (2011). A bayesian foundation for individual learning under uncertainty. Front. Hum. Neurosci..

[41] Mathys C., Daunizeau J., Iglesias S., Diaconescu A.O., Weber L.A.E., Friston K.J., Stephan K.E. (2012). Computational modeling of perceptual inference: A hierarchical Bayesian approach that allows for individual and contextual differences in weighting of input. Int. J. Psychophysiol..

[42] Critchley H.D., Eccles J., Garfinkel S.N. (2013). Interaction between cognition, emotion, and the autonomic nervous system. Handb Clin Neurol..

[43] den Ouden H.E., Daunizeau J., Roiser J., Friston K.J., Stephan K.E. (2010). Striatal prediction error modulates cortical coupling. J. Neurosci..

[44] Rescorla R.A., Wagner A.R., Black A.H., Prokasy W.F. (1972). A theory of Pavlovian conditioning: Variations in the effectiveness of reinforcement. Classical Conditioning II: Current Research and Theory.

[45] Sutton R.S. (1992). Gain adaptation beats least squares?. Proceedings of the Seventh Yale Workshop on Adaptive and Learning Systems.

[46] Cule E., De Iorio M. (2013). Ridge regression in prediction problems: automatic choice of the ridge parameter. Genet. Epidemiol..

[47] Horner A.J., Henson R.N. (2008). Priming, response learning and repetition suppression. Neuropsychologia.

[48] Vossel S., Mathys C., Daunizeau J., Bauer M., Driver J., Friston K.J., Stephan K.E. (2013). Spatial Attention, Precision, and Bayesian Inference: A Study of Saccadic Response Speed. Cereb Cortex..

[49] de Lange F.P., Heilbron M., Kok P. (2018). How do expectations shape perception?. Trends Cogn. Sci..

[50] Aupperle R.L., Melrose A.J., Stein M.B., Paulus M.P. (2012). Executive function and PTSD: disengaging from trauma. Neuropharmacology.

[51] Grupe D.W., Nitschke J.B. (2013). Uncertainty and anticipation in anxiety: an integrated neurobiological and psychological perspective. Nat. Rev. Neurosci..

[52] Pittig A., Treanor M., LeBeau R.T., Craske M.G. (2018). The role of associative fear and avoidance learning in anxiety disorders: Gaps and directions for future research. Neurosci. Biobehav. Rev..

[53] Aylward J., Valton V., Ahn W.-Y., Bond R.L., Dayan P., Roiser J.P., Robinson O.J. (2019). Altered learning under uncertainty in unmedicated mood and anxiety disorders. Nat. Hum. Behav..

[54] Wise T., Michely J., Dayan P., Dolan R.J. (2019). A computational account of threat-related attentional bias. PLoS Comput. Biol..

[55] Hein T.P., de Fockert J., Ruiz M.H. (2020). State anxiety biases estimates of uncertainty and impairs reward learning in volatile environments. NeuroImage.

[56] Press C., Kok P., Yon D. (2020). The Perceptual Prediction Paradox. Trends Cogn. Sci..

[57] Press C., Kok P., Yon D. (2020). Learning to Perceive and Perceiving to Learn. Trends Cogn. Sci..

[58] Corlett P. (2020). Predicting to Perceive and Learning When to Learn. Trends Cogn. Sci..

[59] Cook J.L., Swart J.C., Froböse M.I., Diaconescu A.O., Geurts D.E., Den Ouden (2019). Catecholaminergic modulation of meta-learning. Elife.

[60] Tuross N., Patrick R.L. (1986). Effects of propranolol on catecholamine synthesis and uptake in the central nervous system of the rat. J. Pharmacol. Exp. Ther..

[61] Sara S.J., Bouret S. (2012). Orienting and reorienting: the locus coeruleus mediates cognition through arousal. Neuron.

[62] Sevgi M., Diaconescu A.O., Tittgemeyer M., Schilbach L. (2016). Retraction. Biol. Psychiatry.

[63] Davidson K.W., Alcántara C., Miller G.E. (2018). Selected psychological comorbidities in coronary heart disease: Challenges and grand opportunities. The American psychologist.

[64] Khalsa S.S., Adolphs R., Cameron O.G., Critchley H.D., Davenport P.W., Feinstein J.S., Feusner J.D., Garfinkel S.N., Lane R.D., Mehling W.E., Interoception Summit 2016 participants (2018). Interoception and Mental Health: A Roadmap. Biol. Psychiatry Cogn. Neurosci. Neuroimaging.

[65] Azzalini D., Rebollo I., Tallon-Baudry C. (2019). Visceral Signals Shape Brain Dynamics and Cognition. Trends Cogn. Sci..

[66] Paulus M.P., Feinstein J.S., Khalsa S.S. (2019). An Active Inference Approach to Interoceptive Psychopathology. Annu. Rev. Clin. Psychol..

[67] Owens A.P., Allen M., Ondobaka S., Friston K.J. (2018). Interoceptive inference: From computational neuroscience to clinic. Neurosci. Biobehav. Rev..

[68] Kühnel A., Teckentrup V., Neuser M.P., Huys Q.J.M., Burrasch C., Walter M., Kroemer N.B. (2020). Stimulation of the vagus nerve reduces learning in a go/no-go reinforcement learning task. Eur. Neuropsychopharmacol..

[69] Forte G., De Pascalis V., Favieri F., Casagrande M. (2019). Effects of Blood Pressure on Cognitive Performance: A Systematic Review. Journal of clinical medicine.

[70] Garfinkel S.N., Minati L., Gray M.A., Seth A.K., Dolan R.J., Critchley H.D. (2014). Fear from the heart: sensitivity to fear stimuli depends on individual heartbeats. J. Neurosci..

[71] Allen M., Frank D., Schwarzkopf D.S., Fardo F., Winston J.S., Hauser T.U., Rees G. (2016). Unexpected arousal modulates the influence of sensory noise on confidence. eLife.

[72] Petzschner F.H., Weber L.A., Wellstein K.V., Paolini G., Do C.T., Stephan K.E. (2019). Focus of attention modulates the heartbeat evoked potential. Neuroimage.

[73] Littel M., Kenemans J.L., Baas J.M.P., Logemann H.N.A., Rijken N., Remijn M., Hassink R.J., Engelhard I.M., van den Hout M.A. (2017). The Effects of β-Adrenergic Blockade on the Degrading Effects of Eye Movements on Negative Autobiographical Memories. Biol. Psychiatry.

[74] Hauser T.U., Moutoussis M., Purg N., Dayan P., Dolan R.J. (2018). Beta-Blocker Propranolol Modulates Decision Urgency During Sequential Information Gathering. J. Neurosci..

[75] Bieling P.J., Antony M.M., Swinson R.P. (1998). The State-Trait Anxiety Inventory, Trait version: structure and content re-examined. Behav. Res. Ther..

[76] McDevitt D.G. (1987). Comparison of pharmacokinetic properties of beta-adrenoceptor blocking drugs. Eur. Heart J..

[77] Willenbockel V., Sadr J., Fiset D., Horne G.O., Gosselin F., Tanaka J.W. (2010). Controlling low-level image properties: the SHINE toolbox. Behav. Res. Methods.

[78] Mathys C.D., Lomakina E.I., Daunizeau J., Iglesias S., Brodersen K.H., Friston K.J., Stephan K.E. (2014). Uncertainty in perception and the Hierarchical Gaussian Filter. Front. Hum. Neurosci..

[79] Kasess C.H., Stephan K.E., Weissenbacher A., Pezawas L., Moser E., Windischberger C. (2010). Multi-subject analyses with dynamic causal modeling. Neuroimage.

[80] Hauser T.U., Iannaccone R., Ball J., Mathys C., Brandeis D., Walitza S., Brem S. (2014). Role of the medial prefrontal cortex in impaired decision making in juvenile attention-deficit/hyperactivity disorder. JAMA Psychiatry.

[81] Rigoux L., Stephan K.E., Friston K.J., Daunizeau J. (2014). Bayesian model selection for group studies - revisited. Neuroimage.

[82] Shrout P.E., Fleiss J.L. (1979). Intraclass correlations: uses in assessing rater reliability. Psychol. Bull..

[83] Landis J.R., Koch G.G. (1977). The measurement of observer agreement for categorical data. Biometrics.

[84] Jackson I., Sirois S. (2009). Infant cognition: going full factorial with pupil dilation. Dev. Sci..

[85] Groppe D.M., Urbach T.P., Kutas M. (2011). Mass univariate analysis of event-related brain potentials/fields II: Simulation studies. Psychophysiology.

