# Supplementary material for: The Computational, Pharmacological, and Physiological Determinants of Sensory Learning under Uncertainty
Source: Curr Biol. 2021 Jan 11;31(1):163–172.e4. doi: 10.1016/j.cub.2020.10.043 (PMC7808754; doi:10.1016/j.cub.2020.10.043)
Supplement: Document S1. Figures S1 and S2 and Supplemental References [file mmc1.pdf]

Current Biology, Volume *31*

Supplemental Information

The Computational, Pharmacological, and Physiological Determinants of Sensory Learning under Uncertainty

Rebecca P. Lawson, James Bisby, Camilla L. Nord, Neil Burgess, and Geraint Rees

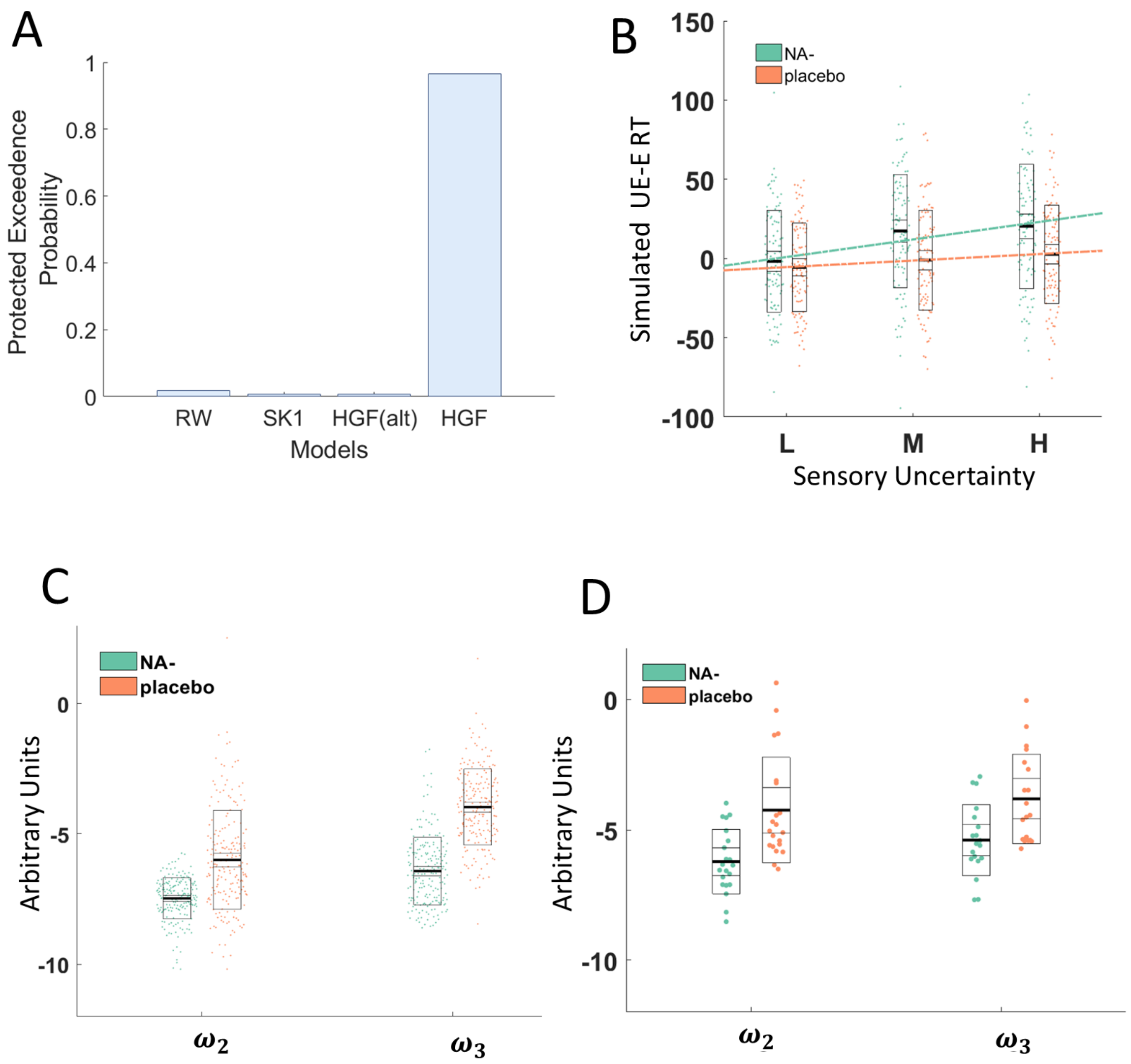

Figure S1: Model Validation, Related to Model Validation section in the STAR methods.
A) Shows the protected exceedance probability (a comparison of Bayesian model evidence) for four different models fit to the data. RW = Rescorla Wagner, SK1 = Sutton K1, HGF (alt) = alternative Hierarchical Gaussian Filter, HGF = Hierarchical Gaussian Filter. See main text for details. B) simulated RTs can recapitulate the primary behavioural result. The parameter estimates (ω_2, ω_3) resulting from model inversion using C) 100 simulations of the mean parameters from the propranolol and placebo groups, and also D) the individual participants parameters for the placebo and propranolol groups. Each datapoint represents the average of 20 simulations.

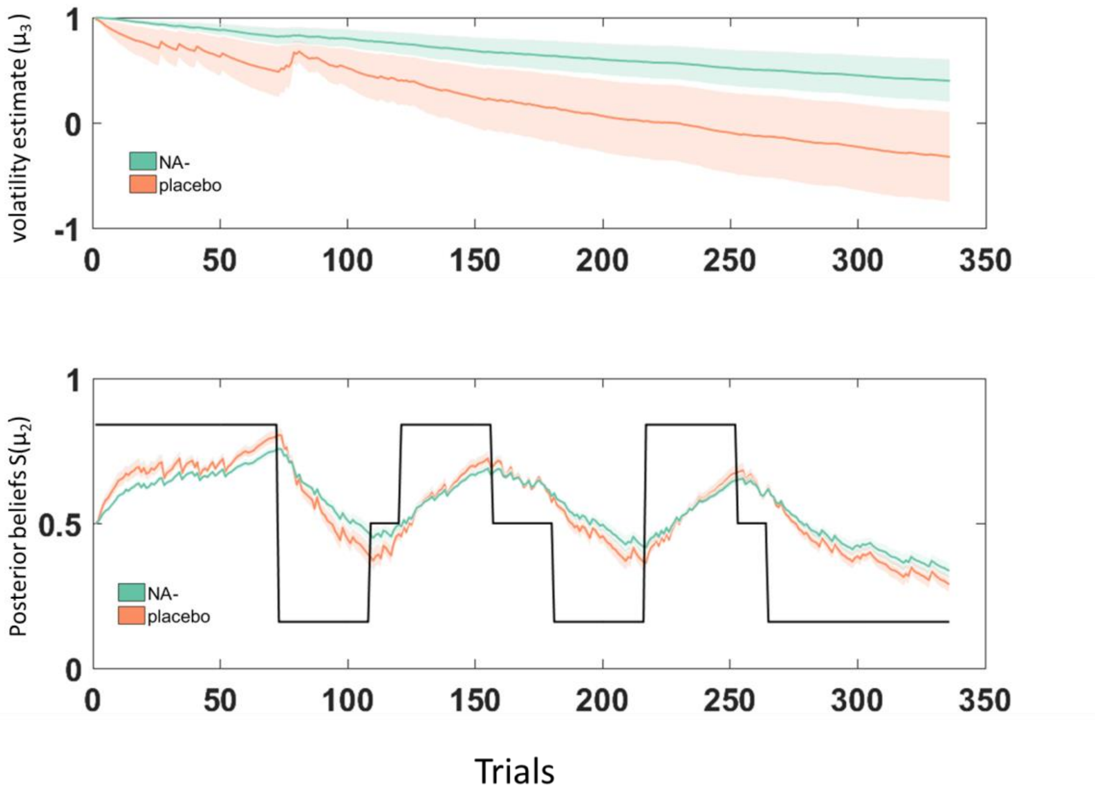

Figure S2: Exploratory analysis of learning trajectories, Related to Figure 4.
Plots show the average µ2 (bottom) and µ3 (top) trajectories estimated from the individual subject-level fits. Note that the shaded error bars represent the standard error of the mean (s.e.m) across the estimates of µ2 and µ3 respectively, not the precision of these beliefs as determined by the HGF. Green lines represent the propranolol group and orange lines represent the placebo group. The thick black line in the bottom panel shows the 'ground truth' changing P(image|tone). In our experimental design we did not systematically manipulate stimulus contingencies across time, which precludes the formulation of clear block-by-block hypotheses about the trajectories of µ2 and µ3 (as in [S1]. On visual expectation, estimates of µ2 appear slower to adjust to the changing stimulus probabilities under propranolol (see also Bayesian Parameter Average plots in Figure 4c). Furthermore, the estimate of µ3 appears lower in the placebo group. However, in an exploratory yet statistically conservative analysis, we used cluster-based permutation tests to assess whether there were differences between the propranolol and placebo groups in the average subject-level estimates of these trajectories across trials in the experiment. In each case, no timepoints were identified in which the groups differed significantly at a cluster-based alpha of 0.05 (2-tailed), 2000 permutations.

Supplemental References

S1. Powers, A.R., Mathys, C., and Corlett, P.R. (2017). Pavlovian conditioning–induced hallucinations result from overweighting of perceptual priors. Science *357*, 596–600.
